# Associations of family health and perceived social support with health literacy among middle-aged and older adults with chronic diseases: a mediation and network analysis

**DOI:** 10.3389/fpubh.2026.1931043

**Published:** 2026-09-23

**Authors:** Huimin Zhang, Kun Pan, Xinyi Zhu, Chenglin Fang, Junhan Liu, Shuaiyou Wang, Zhanyan Han, Jizhi Zhang, Hongru Wang, Chongmei Huang, Minhui Liu

**Affiliations:** 1 School of Nursing, Henan Medical University, Xinxiang, Henan, China; 2 School of Nursing, Ningxia Medical University, Yinchuan, Ningxia, China; 3 Department of Nursing, Xinxiang First People's Hospital, Xinxiang, Henan, China

**Keywords:** family health, health literacy, mediation analysis, middle-aged and older adults with chronic diseases, network analysis, perceived social support

## Abstract

Family and social contexts may play important roles in health literacy among middle-aged and older adults with chronic diseases. This study examined the associations among family health, perceived social support, and health literacy and explored their overall and dimensional relationships using mediation and network analyses. Data were obtained from 1,444 participants in the 2021 Psychology and Behavior Investigation of Chinese Residents. Family health, perceived social support, and health literacy were assessed using the Family Health Scale Short Form, Perceived Social Support Scale, and Health Literacy Survey Questionnaire Short Form 12, respectively. Family health, perceived social support, and health literacy were significantly and positively correlated (*p* < 0.01). Perceived social support demonstrated a statistically significant partial mediating effect in the association between family health and health literacy (indirect effect = 0.1818, 95% CI: 0.1331–0.2307), accounting for 47.38% of the total effect. Network analysis identified Family Social and Emotional Health Processes as the node with the highest strength and Family Support as the node with the highest bridge expected influence. Network structure and global strength did not differ significantly across sex, urban–rural residence, or age groups. These findings highlight the close relationships of family health and perceived social support with health literacy and provide complementary evidence from overall and dimensional perspectives for understanding health literacy among middle-aged and older adults with chronic diseases.

## Introduction

Chronic diseases have become one of the most important public health challenges worldwide. According to the World Health Organization, noncommunicable diseases caused at least 43 million deaths in 2021, accounting for the vast majority of non-pandemic-related deaths globally (1). With the acceleration of population ageing worldwide, health issues among older adults have received increasing attention (2, 3). In China, the probability of dying from the four major noncommunicable diseases—cardiovascular diseases, cancer, diabetes, and chronic respiratory diseases—among people aged 30–70 years is 15.9% (4). Data from the National Bureau of Statistics show that, by the end of 2025, the population aged 60 years and older in China had reached 323.38 million, accounting for 23.0% of the total population, among whom 223.65 million were aged 65 years and older, accounting for 15.9% (5). Against the combined background of population ageing and the burden of chronic diseases, the need for health promotion and management among middle-aged and older adults with chronic diseases has become increasingly prominent (6). During middle and older adulthood, chronic disease management becomes increasingly embedded in long-term daily care, family involvement, and the use of social and health-related resources. Therefore, understanding how family and social resources are associated with patients’ health management capacity is important for promoting healthy ageing and supporting long-term chronic disease management.

Health literacy is an important foundation for patients with chronic diseases to process health information and engage in health management. It refers to individuals’ ability to access, understand, appraise, and apply health information to make decisions related to healthcare, disease prevention, and health promotion (7). Chronic disease management often involves long-term medication use, symptom recognition, daily decision-making, and behavioral adjustment; therefore, patients’ ability to access, understand, appraise, and apply health information may be associated with the effectiveness of chronic disease self-management (7, 8). For middle-aged and older adults with chronic diseases, long-term disease management requires continuous engagement with health information and healthcare services, as well as ongoing health-related decision-making, making health literacy particularly relevant. Previous studies have shown that higher levels of health literacy are associated with more positive illness perceptions, treatment beliefs, and self-management behaviors (9, 10). In addition, interventions focusing on health literacy may improve certain management outcomes among patients with chronic diseases and have increasingly become an important focus of interventions for older adults (11–14). According to the integrated model of health literacy, health literacy is not only an individual-level information-processing capacity but is also shaped by personal characteristics, social contexts, and environmental resources (7). Therefore, understanding health literacy among middle-aged and older adults with chronic diseases requires attention not only to individual capacities but also to the family and social contexts in which they live and the support resources available to them (15).

The family is the primary living environment for middle-aged and older adults with chronic diseases and an important setting in which disease management and health maintenance continuously take place. In recent years, family health has increasingly been recognized as an important family-level concept for understanding individual health behaviors and health outcomes. Family health is defined as “a resource at the level of the family unit that develops from the intersection of the health, interactions, and capacities of each family member, as well as the family’s physical, social, emotional, economic, and medical resources” (16). As one of the most immediate and enduring health contexts across the life course, the family can continuously shape family members’ health behaviors and outcomes through continuity of care, daily health norms, emotional connections, and resource coordination (17, 18). Conceptually, family health is a multidimensional family-level construct that encompasses not only relationships and interactions among family members but also family healthy lifestyles, health-related resources, and external social support available to the family. More specifically, it reflects communication and emotional connections among family members, the family’s capacity to jointly maintain health-related behaviors, the availability and mobilization of health resources, and the ability to obtain support from external social networks. Thus, family health does not represent a single aspect of family functioning or support; rather, it reflects the family’s overall resource state for maintaining and promoting the health of its members (16, 17). Previous research has shown that higher levels of family health are associated with higher levels of perceived social support and self-efficacy, and that perceived social support partially mediates the relationship between family health and self-efficacy (19). These findings suggest that favorable family health may be associated with patients’ perception and utilization of support resources and may provide a family-based foundation for their ability to access, understand, appraise, and apply health information.

In the long-term management of chronic diseases, perceived social support is an important psychosocial resource closely associated with patients’ health management and primarily reflects individuals’ subjective perceptions of support from family members, friends, and significant others. Such support may include emotional comfort, practical assistance, and health-related guidance, thereby helping patients cope with the ongoing demands of chronic disease management (20). For middle-aged and older adults with chronic diseases, perceived social support may be particularly relevant to sustained health management. Patients with higher levels of social support may obtain explanations of health information, assistance with communication with healthcare professionals, and support for health-related decision-making through their social networks, thereby facilitating their ability to access, understand, appraise, and apply health information (21). Previous studies have shown that social support is closely associated with health literacy and related health outcomes among older adults with chronic diseases (22). Empirical evidence has also supported an association between family health and perceived social support (19, 23). It should be noted that although family health and perceived social support are related, they are conceptually distinct. Family health refers to the health-related resources and functioning of the family as an integrated system, whereas perceived social support refers to an individual’s subjective perception of support from family members, friends, and significant others. From the perspective of stress and coping theory, long-term chronic disease management can be viewed as an ongoing stressful context in which patients must continuously appraise and mobilize resources, including family and social support, to cope with disease-management demands (24). Therefore, perceived social support may represent an important psychosocial pathway linking family health and health literacy: patients with better family health may be more likely to perceive and utilize available support resources, and such perceived support may in turn be associated with their health information-processing and health-related decision-making capacities.

Previous studies have indicated that family health, perceived social support, and health literacy are all associated with chronic disease management (19, 25, 26). However, several gaps remain in the existing literature. First, previous health literacy research has primarily focused on individual-level factors, such as self-efficacy, illness perceptions, and health behaviors, whereas comparatively limited attention has been paid to family health as a broader family-level resource. Second, although family health is closely associated with perceived social support and individuals’ health management capacity, direct evidence remains limited regarding whether perceived social support plays a statistical mediating role in the association between family health and health literacy. Third, previous studies have predominantly used variable-centered approaches, such as correlation analysis, regression analysis, and structural equation modeling. Although these methods are useful for examining overall associations among variables, they provide relatively limited information about how specific dimensions of different constructs are interconnected. In contrast, network analysis can represent the dimensions of family health, perceived social support, and health literacy as nodes, characterize the conditional associations among these dimensions, and identify central and bridge nodes within the network, thereby complementing traditional variable-centered approaches at a more fine-grained dimensional level (27–29). Accordingly, combining mediation and network analyses can provide complementary evidence on the relationships among these three constructs from two perspectives: overall statistical associations and more detailed dimensional association structures.

Accordingly, this study had three objectives: (1) to examine the associations among family health, perceived social support, and health literacy; (2) to examine the statistical mediating role of perceived social support in the association between family health and health literacy; and (3) to use network analysis to identify central and bridge dimensions across the three constructs and to further explore whether network structure and global strength differed by sex, urban–rural residence, and age group. The following hypotheses were proposed: H1: family health, perceived social support, and health literacy are significantly and positively correlated; and H2: perceived social support has a statistically significant mediating effect in the association between family health and health literacy. The findings may provide evidence to inform health literacy promotion and family support-related nursing interventions for middle-aged and older adults with chronic diseases. The remainder of this paper describes the study methods, presents the results of the correlation, mediation, and network analyses, and discusses the findings.

## Materials and methods

### Data source and participants

Data for this study were obtained from the 2021 Psychology and Behavior Investigation of Chinese Residents (PBICR) (30). The study protocol was approved by the Medical Ethics Committee of Jinan University (JNUKY-2021-018). The survey used a multistage sampling method and covered 120 cities across 32 provincial-level regions in China. The survey was administered by uniformly trained investigators, and participants completed the questionnaire after providing informed consent. The questionnaire covered sociodemographic characteristics, personal health status, family-related information, social environment, and psychological and health behavior-related measures. In 2021, a total of 11,031 valid responses were collected.

Participants were further screened from this database according to the study objectives. The inclusion criteria were: (1) patients with chronic diseases aged 41–85 years; and (2) complete data on the key study variables and logically consistent questionnaire responses. After screening according to these criteria, 1,444 eligible patients with chronic diseases were included in the final analysis. The participant selection process is shown in Figure 1.

**Figure 1:**
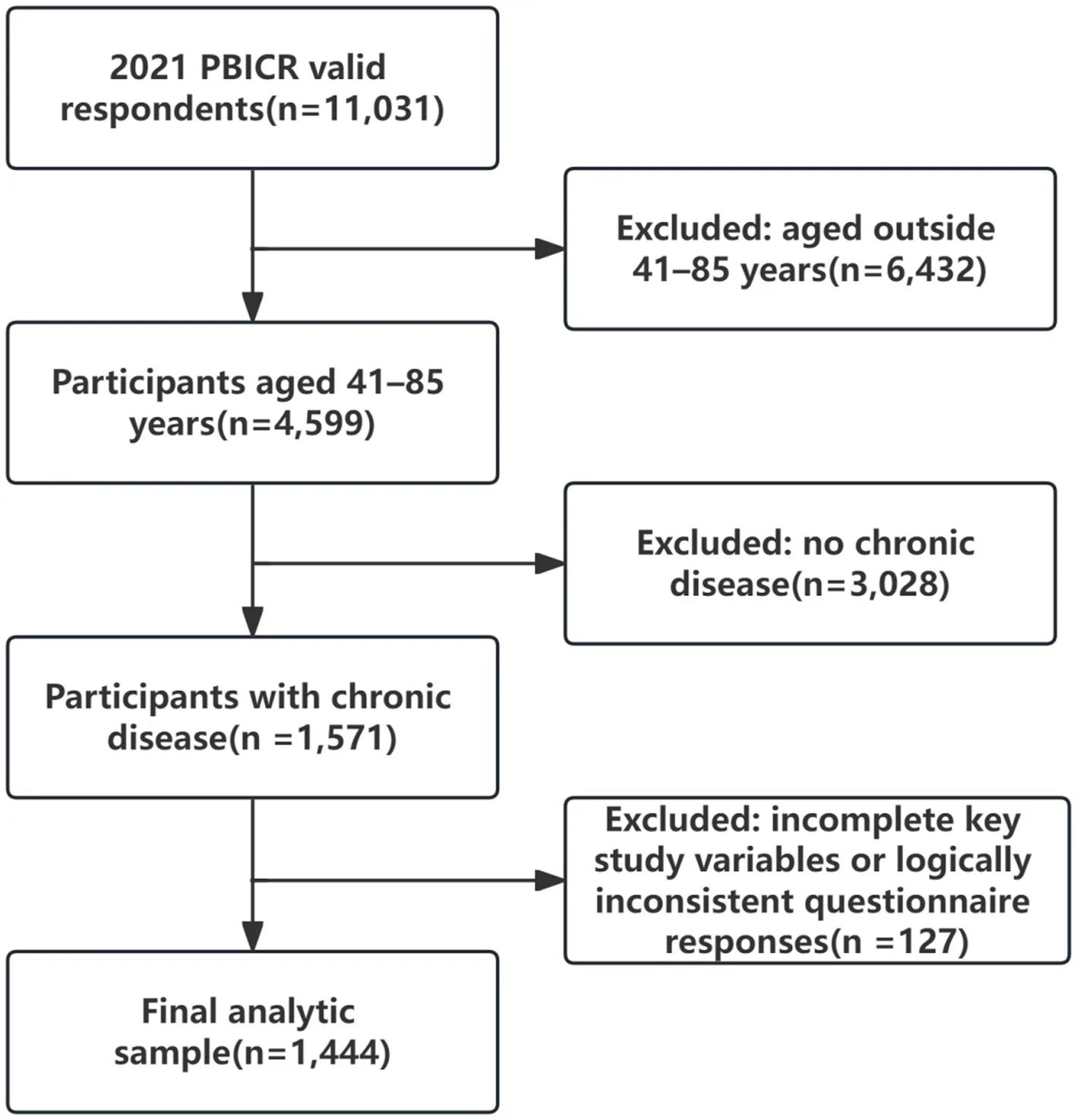
Flowchart of participant selection from the 2021 PBICR database.

## Research instruments

### Sociodemographic information

Age, sex, residence, marital status, and educational attainment were extracted from the questionnaire as sociodemographic variables. In the original database, age was recorded in nine age categories ranging from 41–45 to 81–85 years. For descriptive statistics and covariate adjustment in the mediation analysis, the midpoint of each age category was used to represent age. To facilitate covariate adjustment in the mediation analysis, sex was recoded as male = 0 and female = 1, residence as rural = 0 and urban = 1, and marital status was dummy-coded with married participants as the reference category. Educational attainment was consolidated into four categories—primary school or below, junior high school, senior high school/technical secondary school, and junior college or above—and was dummy-coded with primary school or below as the reference category. For subgroup network comparisons, participants were additionally classified into a middle-aged group (41–59 years) and an older group (60–85 years).

### Family health scale–short form (FHS-SF)

The Family Health Scale–Short Form (FHS-SF), developed by Crandall et al. (17), was used to assess family health functioning and was translated into Chinese by Wang et al. (31). The scale consists of 10 items across four dimensions: family social and emotional health processes, family healthy lifestyle, family health resources, and family external social support. Each item is rated on a 5-point Likert scale ranging from “strongly disagree” to “strongly agree” (1–5 points), with Items 6, 9, and 10 reverse scored. A higher total score indicates a higher level of family health. In the present study, the Cronbach’s α coefficient for this scale was 0.848.

### Perceived social support scale (PSSS)

The Perceived Social Support Scale (PSSS), developed by Zimet et al. (32), was used to assess perceived social support. The PSSS comprises 12 items across three dimensions: family support, friend support, and significant other support, with four items in each dimension. Items are rated on a 7-point Likert scale ranging from “very strongly disagree” to “very strongly agree” (1–7 points). Higher scores indicate greater perceived social support. In the present study, the Cronbach’s α coefficient for this scale was 0.941.

### Health literacy survey questionnaire short form 12 (HLS-SF12)

The Health Literacy Survey Questionnaire Short Form 12 (HLS-SF12), developed by Duong et al. (33), was used to assess health literacy. The scale consists of 12 items across three dimensions: health care, disease prevention, and health promotion. Each item is rated using a 4-point Likert scale. Higher scores indicate a higher level of health literacy. In the present study, the Cronbach’s α coefficient for this scale was 0.916.

### Statistical analysis

Data management and descriptive statistical analyses were performed using SPSS version 26.0. Pearson correlation analysis was used to examine the correlations among family health, perceived social support, and health literacy. Multicollinearity among the predictors was assessed using tolerance values and variance inflation factor (VIF) values. The PROCESS macro developed by Hayes (Model 4) was subsequently used to examine the statistical mediating role of perceived social support in the association between family health and health literacy. Indirect effects were tested using 5,000 bootstrap resamples, and an indirect effect was considered statistically significant when its 95% confidence interval did not include zero.

Network analysis was conducted using R. The EBICglasso model was used to estimate a partial correlation network among the dimensions of family health, perceived social support, and health literacy (29), and network estimation and visualization were performed using the “bootnet” and “qgraph” packages. Each dimension was treated as a node, and conditional associations between nodes were represented as edges. Edge thickness reflected the strength of the association, with thicker edges indicating stronger associations, whereas edge color reflected the direction of the association, with blue indicating positive associations and red indicating negative associations. Node strength was used to assess centrality, and centrality estimates were standardized as Z-scores. Because closeness and betweenness centrality may show relatively low stability (34, 35), only strength centrality was reported in the present study. In addition, the bridge function in the“networktools”package was used to calculate 1-step bridge expected influence to assess the cross-community connectivity of nodes across different construct communities (28).

The accuracy and stability of the network estimates were evaluated using the “bootnet” package (29). A nonparametric bootstrap procedure was used to estimate 95% confidence intervals for edge weights to assess the accuracy of edge-weight estimates, with narrower confidence intervals indicating greater precision (36). A case-dropping bootstrap procedure was used to assess the stability of centrality estimates, quantified using the correlation stability coefficient (CS-C). A CS-C greater than 0.25 is generally considered acceptable, whereas a value greater than 0.50 is preferable (29). Bootstrap-based difference tests were also conducted to examine whether edge weights and node centrality estimates differed significantly from one another (29). Finally, the Network Comparison Test (NCT) was conducted using the “NetworkComparisonTest”package (37) to evaluate network structure invariance and global strength invariance across subgroups. Permutation tests with 1,000 permutations were used to compare network structure and global strength between male and female participants, rural and urban residents, and the middle-aged (41–59 years) and older (60–85 years) groups (38).

## Results

### Demographic characteristics

The demographic characteristics of all participants are presented in Table 1. Based on the midpoints of the age categories, the mean age of the participants was 59.09 years (SD = 12.07). Of the 1,444 participants, 44.9% were female (*n* = 649) and 55.1% were male (*n* = 795). Regarding residence, 68.2% lived in urban areas (*n* = 985) and 31.8% lived in rural areas (*n* = 459). Most participants were married (86.5%, *n* = 1,249), followed by widowed (9.1%, *n* = 132), divorced (2.6%, *n* = 38), and unmarried participants (1.7%, *n* = 25). With respect to educational attainment, 28.1% had completed primary school or below (*n* = 406), 21.5% had completed junior high school (*n* = 310), 20.2% had completed senior high school or technical secondary school (*n* = 292), and 30.2% had completed junior college or above (*n* = 436).

**Table 1:** Demographic characteristics of participants.

Variables	*n* (%)
Sex	
Female	649(44.9%)
Male	795(55.1%)
Residence	
Urban	985(68.2%)
Rural	459(31.8%)
Marital status	
Married	1,249(86.5%)
Unmarried	25(1.7%)
Divorced	38(2.6%)
Widowed	132(9.1%)
Educational level	
Primary school or below	406(28.1%)
Junior high school	310(21.5%)
Senior high school/technical secondary school	292(20.2%)
Junior college or above	436 (30.2%)

### Correlation analysis

Pearson correlation analysis was conducted to examine the relationships among family health, perceived social support, and health literacy (Table 2). Family health was significantly and positively correlated with perceived social support (*r* = 0.587, *p* < 0.01) and health literacy (*r* = 0.338, *p* < 0.01). Perceived social support was also significantly and positively correlated with health literacy (*r* = 0.359, *p* < 0.01).

**Table 2:** Correlations among family health, perceived social support, and health literacy.

Variables	M	SD	1	2	3
1. Family health	38.76	6.39	1		
2. Perceived social support	59.98	11.71	0.587**	1	
3. Health literacy	31.25	7.90	0.338**	0.359**	1

** *p* < 0.01.

### Mediation analysis

In the regression model with health literacy as the outcome variable, multicollinearity diagnostics showed that tolerance values ranged from 0.434 to 0.985 and VIF values ranged from 1.015 to 2.303, indicating no evidence of problematic multicollinearity among the predictors. After adjustment for sex, age, marital status, residence, and educational attainment, mediation analysis was performed using Model 4 of the SPSS PROCESS macro (Figure 2). The total effect of family health on health literacy was statistically significant [total effect = 0.3837, 95% CI (0.3270, 0.4404)]. After perceived social support was included in the model, the direct effect of family health on health literacy remained statistically significant [direct effect = 0.2018, 95% CI (0.1346, 0.2691)], consistent with partial mediation. Bootstrap analysis showed that the indirect effect through perceived social support was 0.1818 [95% CI (0.1331, 0.2307)]. Because the confidence interval did not include zero, the indirect effect was statistically significant. The indirect effect accounted for 47.38% of the total effect, indicating a statistically significant partial mediating role of perceived social support in the association between family health and health literacy. Specifically, family health was significantly and positively associated with perceived social support [B = 1.0609, 95% CI (0.9823, 1.1394)], and perceived social support was significantly and positively associated with health literacy [B = 0.1714, 95% CI (0.1350, 0.2078)].

**Figure 2:**
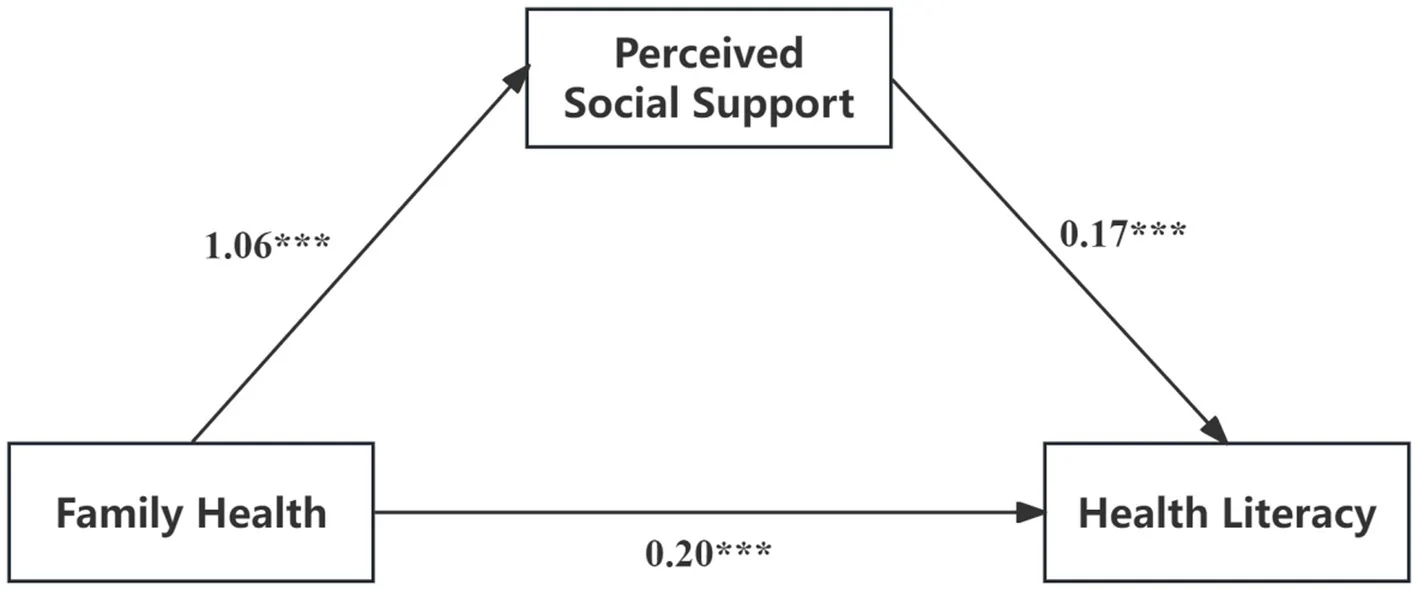
Mediation model of perceived social support in the relationship between family health and health literacy. Note: Values in the figure are unstandardized path coefficients; *** *p* < 0.001.

### Network analysis

The network of family health, perceived social support, and health literacy consisted of 10 nodes and 32 nonzero edges, representing 71% of all possible edges (Figure 3). Family Social and Emotional Health Processes (FHS-SF1) had the highest node strength, followed by Other Support (PSSS3) and Disease Prevention (HLS-SF2) (Figure 4). Higher node strength indicates stronger overall conditional associations between a given dimension and the other dimensions in the network; therefore, these dimensions showed relatively high overall connectivity within the network. Bridge expected influence analysis showed that Family Support (PSSS1) had the highest bridge expected influence, followed by Family External Social Supports (FHS-SF4) and Family Social and Emotional Health Processes (FHS-SF1) (Figure 5). Higher bridge expected influence indicates stronger overall connectivity with nodes belonging to other construct communities; thus, these dimensions showed relatively prominent cross-community connectivity among family health, perceived social support, and health literacy.

**Figure 3:**
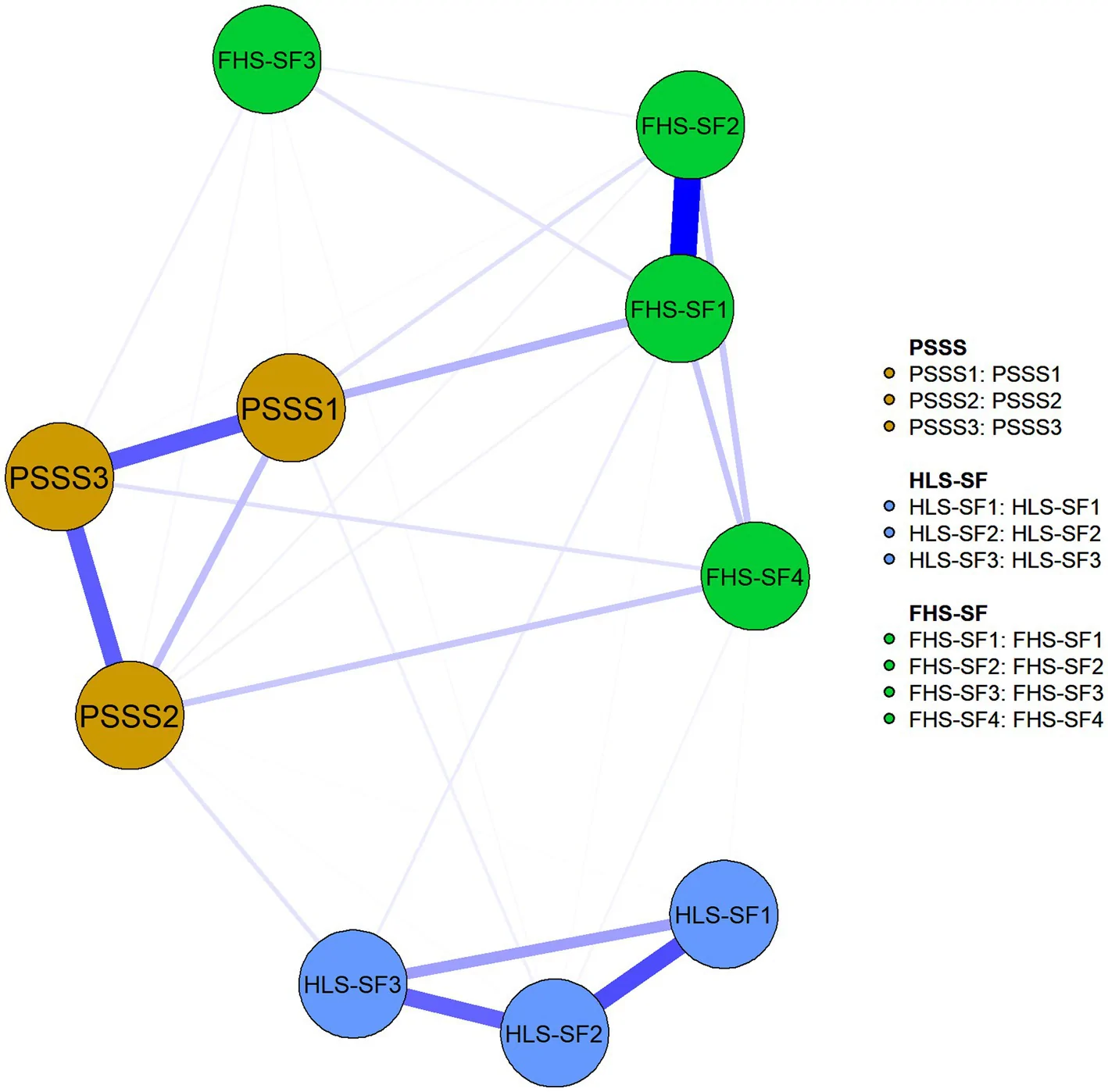
Network structure of family health, perceived social support, and health literacy among middle-aged and older adults with chronic diseases. Note: PSSS1: Family support; PSSS2: Friend support; PSSS3: Other support. HLS-SF1: Health care; HLS-SF2: Disease prevention; HLS-SF3: Health promotion. FHS-SF1: Family social and emotional health processes; FHS-SF2: Family healthy lifestyle; FHS-SF3: Family health resources; FHS-SF4: Family external social supports.

**Figure 4:**
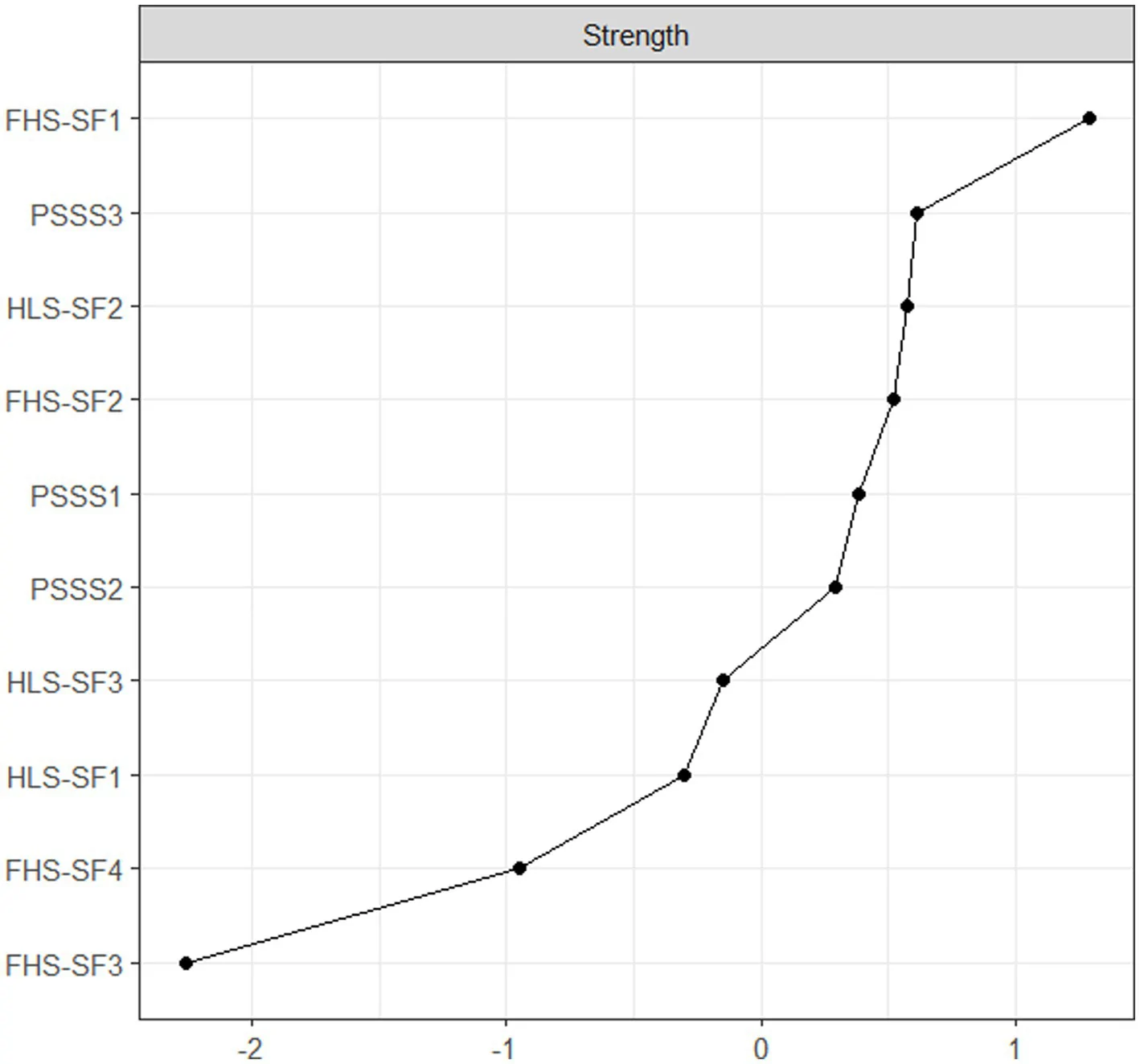
Node strength plot of family health, perceived social support, and health literacy among middle-aged and older adults with chronic diseases.

**Figure 5:**
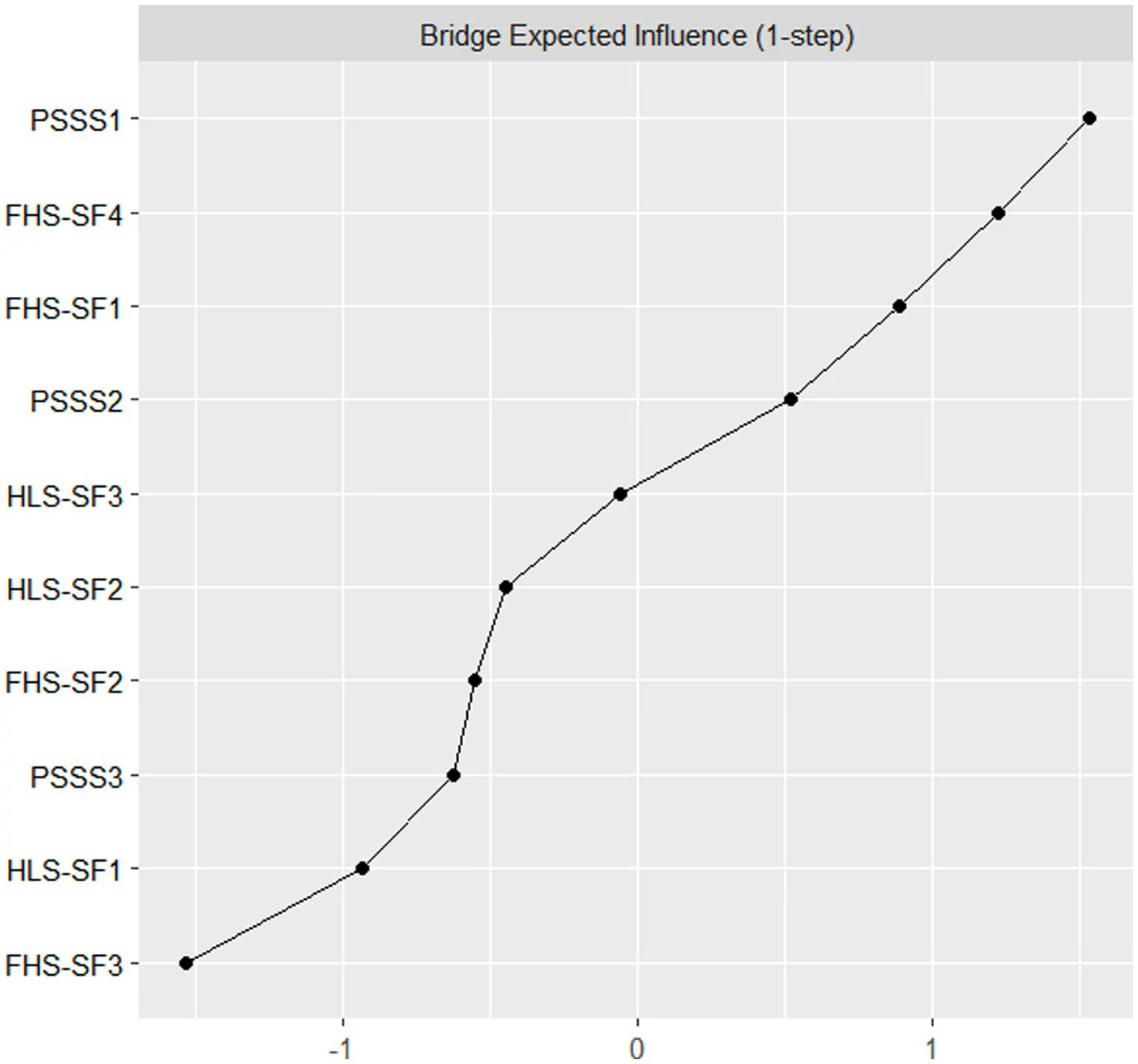
Bridge expected influence of nodes for family health, perceived social support, and health literacy among middle-aged and older adults with chronic diseases.

The case-dropping bootstrap procedure yielded a correlation stability coefficient (CS-C) of 0.75, indicating high stability of the centrality estimates (Figure 6). Bootstrap difference tests indicated statistically significant differences among some edge weights (See Additional file, Supplementary Figure S1). In addition, the nonparametric bootstrap procedure showed relatively narrow 95% confidence intervals for the edge weights, suggesting good accuracy of the edge-weight estimates (See Additional file, Supplementary Figure S2).

**Figure 6:**
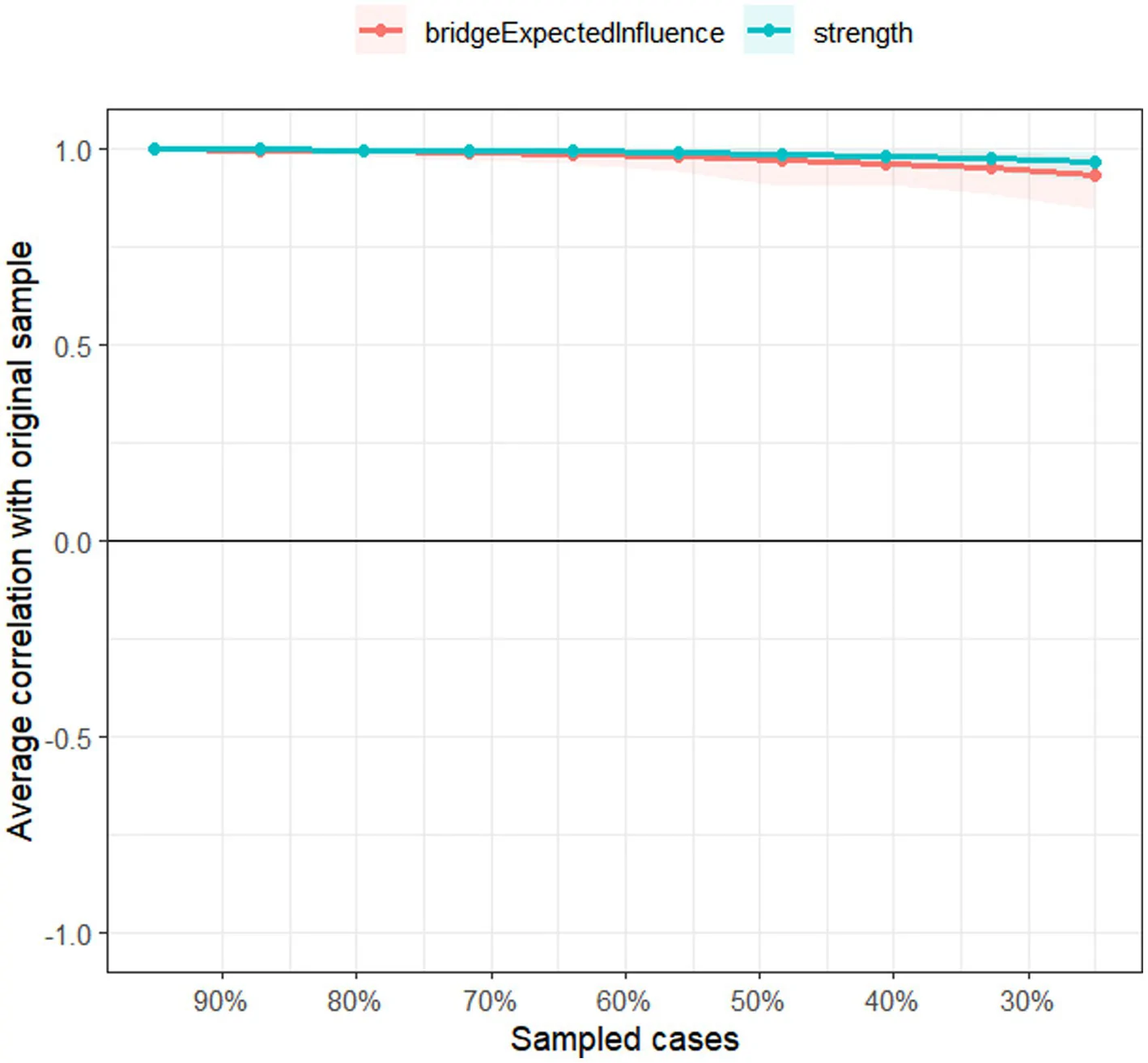
Stability of strength and bridge expected influence in the network of family health, perceived social support, and health literacy among middle-aged and older adults with chronic diseases.

Network comparison analyses showed no statistically significant difference in global network strength between male and female participants (4.335 vs. 4.121, S = 0.213, *p* = 0.208), and the network structure invariance test likewise showed no statistically significant difference between the two groups (M = 0.102, *p* = 0.507). Similarly, no statistically significant difference in global network strength was observed between urban and rural participants (4.373 vs. 4.101, S = 0.272, *p* = 0.107), and no statistically significant difference in network structure was found (M = 0.086, *p* = 0.861). The age-group comparison also showed no statistically significant difference in global network strength between the middle-aged group (41–59 years) and the older group (60–85 years) (4.416 vs. 4.214, S = 0.201, *p* = 0.233), with no statistically significant difference in network structure between the two age groups (M = 0.122, *p* = 0.228) (See Additional file, Supplementary Figure S3).

## Discussion

This study examined the relationships among family health, perceived social support, and health literacy among middle-aged and older adults with chronic diseases and further explored their associations and structural characteristics using mediation and network analyses. The results showed significant positive correlations among family health, perceived social support, and health literacy, and perceived social support demonstrated a statistically significant partial mediating effect in the association between family health and health literacy. Network analysis further showed that Family Social and Emotional Health Processes (FHS-SF1), Other Support (PSSS3), and Disease Prevention (HLS-SF2) had relatively high node strength. Bridge expected influence analysis showed that Family Support (PSSS1), Family External Social Supports (FHS-SF4), and Family Social and Emotional Health Processes (FHS-SF1) had relatively high bridge expected influence. These findings indicate that family health, perceived social support, and health literacy are associated not only at the overall construct level but may also form an interconnected structure at the dimensional level.

This study found that both family health and perceived social support were significantly and positively associated with health literacy, suggesting that these three constructs are not isolated from one another but are jointly embedded in the health management of middle-aged and older adults with chronic diseases. Previous studies have shown that family health is closely associated with perceived social support and self-efficacy (19), while social support has also been associated with family health and family functioning (23), health literacy (22), and self-management behaviors among patients with chronic diseases (26). In the Chinese sociocultural context, families generally exhibit a strong family orientation and sense of collective responsibility, with family members often maintaining one another’s health through mutual support and shared participation within the family (39). Family involvement has also been shown to be closely associated with self-management and related health outcomes among patients with chronic diseases (40). Therefore, better family health may provide a more supportive family context in which patients can obtain assistance, communicate health information, and participate in health management. Compared with previous studies that have primarily focused on the relationships of family health with self-efficacy or self-management-related outcomes, the present study further identified a significant association between family health and health literacy, extending existing evidence on the relationship between family-level resources and individual health management capacities. In the context of population ageing and long-term chronic disease management, this finding further highlights the importance of considering the family and social support environments in which middle-aged and older adults manage their health. The mediation analysis further showed that perceived social support had a statistically significant partial mediating effect in the association between family health and health literacy. This finding extends the work of Luo et al. (19), who reported a mediating role of perceived social support in the relationship between family health and self-efficacy, by suggesting that perceived social support may also be involved in the statistical indirect association between family health and health literacy. Previous research has shown that higher levels of social support are associated with more active engagement in self-management among older adults with chronic diseases (41). Studies of patients with chronic diseases and their family caregivers have also indicated that self-management is not carried out entirely by patients alone; family members often remain involved in self-management decision-making, task sharing, and caregiving support (42, 43). From the perspectives of the integrated model of health literacy and stress and coping theory, family and social environments can provide potentially available support resources, while patients’ perceptions and mobilization of these resources may be associated with their ability to cope with the ongoing demands of chronic disease management and to access, understand, and apply health information (7, 24). Thus, better family health may be associated with patients perceiving greater understanding, care, and assistance in daily life, and these positive perceptions of support may in turn be associated with greater confidence in coping with disease, a stronger sense of stability, and better health information-processing capacity.

Network analysis provided further insight into the dimensional association structure among family health, perceived social support, and health literacy. Family Social and Emotional Health Processes (FHS-SF1) had the highest node strength, indicating that this dimension had strong overall conditional associations with other dimensions in the network. Emotional connections, communication, and interactions among family members constitute an important family context for long-term chronic disease management, and previous research has shown that family behaviors and communication patterns are closely associated with self-management and related health outcomes among patients with chronic diseases (44). Therefore, positive family social and emotional processes may provide an important relational context for understanding the associations among multiple dimensions of family health, perceived support, and health literacy. *Other Support (PSSS3)* also showed relatively high node strength, indicating that support from significant others beyond family and friends had relatively high connectivity within the overall network. Research among older patients with chronic respiratory diseases has shown that both online and offline social networks may play a role in illness management (45). In addition, previous studies have indicated that external forms of support, such as peer support, can provide patients with chronic conditions with resources including social connectedness, experiential knowledge, and ongoing support (46). Therefore, stable support from outside the family may be broadly associated with dimensions of family health and health literacy and may provide an additional perspective for understanding the long-term health management context of middle-aged and older adults with chronic diseases. *Disease Prevention (HLS-SF2)* also demonstrated relatively high node strength, which may reflect the importance of prevention-related health information-processing capacity in long-term chronic disease management. For middle-aged and older adults with chronic diseases, disease management involves not only treatment of existing conditions but also prevention of complications, symptom monitoring, risk control, and lifestyle modification. Previous research has shown that health literacy is closely associated with health outcomes among older adults with chronic diseases (47), while studies among older populations have further identified significant associations between health literacy and certain preventive health behaviors (48). Taken together, the capacity to process information related to disease prevention and risk management may represent an important foundation for maintaining long-term health management among middle-aged and older adults with chronic diseases.

Bridge expected influence analysis showed that *Family Support (PSSS1)* had the highest bridge expected influence, indicating that patients’ perceived family support had relatively strong cross-community associations with relevant dimensions in other construct communities. This finding further illustrates both the conceptual distinction and the connection between family health and perceived social support. Family health reflects the overall resources and functioning of the family, whereas Family Support reflects individuals’ subjective perceptions of support received from family members. For patients with chronic diseases, the family is an important social system through which daily care, emotional support, and resource coordination are obtained (19). The role of the family in chronic disease management is not limited to providing practical care but also includes participation in specific management tasks, such as information exchange, medication management, and transitions of care (49). Therefore, patients’ perceptions of family support may help explain the links between family system resources and health information comprehension, participation in health-related decision-making, and the maintenance of health behaviors. *Family External Social Supports (FHS-SF4)* also showed relatively high bridge expected influence, indicating that the family’s capacity to connect with external support systems had relatively strong connectivity across different constructs. Chronic disease management does not occur solely within the family but requires continuing connections between families and communities, healthcare systems, and other external support networks. In recent years, increasing attention has been paid to integrating family-level factors, processes, and outcomes into chronic disease prevention and management to promote more comprehensive family support services (50, 51). Therefore, Family External Social Supports may reflect the family’s capacity to mobilize community, healthcare, and social support resources and may provide an important perspective for understanding the links between family system resources and patients’ individual health management capacities. *Family Social and Emotional Health Processes (FHS-SF1)* also showed relatively high bridge expected influence. Notably, this dimension had both the highest node strength and relatively strong cross-community connectivity, further indicating that emotional interactions and relational processes within the family are closely associated with multiple dimensions across the overall network. Previous research has emphasized that the family should be understood as a system and a social unit; in chronic disease management, self-care is not simply the sum of individual behaviors performed by family members but instead unfolds through family interactions, role allocation, and relational experiences (52). Family involvement in self-care may have beneficial effects on some patient outcomes, particularly in interventions involving family-centered approaches, active learning strategies, and appropriate follow-up (53). Among older adults with chronic diseases, stronger overall family support has also been associated with living with family members and maintaining better family relationships (54). Taken together, positive social and emotional processes within the family may represent an important link among family support resources, external support conditions, and patients’ health management capacities, whereas weaker emotional responsiveness or poorer relational interactions may be associated with lower levels of perceived support and health management capacity.

Network comparisons across sex, urban–rural residence, and age subgroups showed no statistically significant differences in network structure or global strength. These findings indicate that no statistically significant subgroup differences in the overall network structure or connectivity were observed in the present sample. Importantly, this does not imply that patients of different sexes, urban–rural residence, or age groups had identical levels of the study variables; rather, the patterns of associations among family health, perceived social support, and health literacy did not show statistically significant structural differences across these subgroups. One possible explanation is that middle-aged and older adults with chronic diseases generally require family involvement, social support, and access to and processing of health information during long-term disease management.

Overall, the findings suggest that efforts to promote health literacy among middle-aged and older adults with chronic diseases should not focus solely on individual knowledge and information comprehension but should also consider family health and social support resources. In nursing practice, family health assessment, family communication guidance, caregiver health education, and linkage to community resources may be incorporated to support patients in better perceiving and utilizing available family and social support resources. Health education may also place greater emphasis on disease prevention, symptom monitoring, appropriate medication use, and lifestyle modification to support patients’ long-term chronic disease management.

### Strengths and limitations

This study was based on a relatively large survey dataset covering multiple regions of China and combined mediation and network analyses to examine the relationships among family health, perceived social support, and health literacy from both overall and dimensional association perspectives. However, several limitations should be acknowledged. First, the cross-sectional design and reliance on self-reported data limit causal inference and may introduce reporting bias. Second, although several sociodemographic variables were controlled for, unmeasured factors, such as access to healthcare services and health information and family history of chronic disease, may still have resulted in residual confounding. In addition, network analysis conducted at the scale-dimension level may not capture more fine-grained item-level associations. Furthermore, central and bridge nodes in the network reflect structural associations among variable dimensions and should not be interpreted as evidence of causal relationships or directly regarded as intervention targets. Future studies could adopt longitudinal designs and incorporate a broader range of individual-, family-, and healthcare resource-related factors to further examine the stability of these associations and their applicability across different chronic disease populations.

## Conclusion

This study showed significant positive associations among family health, perceived social support, and health literacy among middle-aged and older adults with chronic diseases, and perceived social support demonstrated a statistically significant partial mediating effect in the association between family health and health literacy. Network analysis further showed that Family Social and Emotional Health Processes, Other Support, and Disease Prevention had relatively high node strength, whereas Family Support, Family External Social Supports, and Family Social and Emotional Health Processes had relatively high bridge expected influence. In addition, no statistically significant differences in network structure or global strength were observed across sex, urban–rural residence, or age groups. Overall, this study further highlights the close relationships of family health and social support resources with health literacy among middle-aged and older adults with chronic diseases from both overall and dimensional association perspectives and provides evidence to inform family support-oriented health literacy promotion and long-term chronic disease management. Future studies should further examine the stability and applicability of these associations across different chronic disease types and care contexts.

## Data Availability

The data analyzed in this study were obtained from the Psychology and Behavior Investigation of Chinese Residents (PBICR) database through an application and approval process. The data are not publicly available, and access is subject to the PBICR data access policies and approval procedures. Further information on data access and application procedures is available on the official PBICR website: https://www.x-mol.com/groups/pbicr.
