# Supplementary material for: Associations of family health and perceived social support with health literacy among middle-aged and older adults with chronic diseases: a mediation and network analysis
Source: Front Public Health. 2026 Sep 23;14:1931043. doi: 10.3389/fpubh.2026.1931043 (PMC13641992; doi:10.3389/fpubh.2026.1931043)
Supplement: Supplementary file 1 [file Supplementary_file_1.docx]

**Supplementary Figure S1.Bootstrapped stability test for edge-weight.**

**Note: PSSS1: Family Support; PSSS2: Friend Support; PSSS3: Other Support.**

**HLS-SF1: Health Care; HLS-SF2: Disease Prevention; HLS-SF3: Health Promotion.**

**FHS-SF1: Family social and emotional health processes; FHS-SF2: Family healthy lifestyle; FHS-SF3: Family health resources; FHS-SF4: Family external social supports.**

**Supplementary Figure S2.Bootstrapped confidence intervals of edge weights.**

**Supplementary Figure S3. Network comparisons between male and female participants, urban and rural residents, and middle-aged and older groups .**
